# Immobilization of Antibodies by Genetic Fusion to a Fungal Self-Assembling Adhesive Protein

**DOI:** 10.3389/fmolb.2021.725697

**Published:** 2021-10-19

**Authors:** Ilaria Stanzione, Daniel Izquierdo-Bote, María Begoña González García, Paola Giardina, Alessandra Piscitelli

**Affiliations:** ^1^ Dipartimento di Scienze Chimiche, Naples, Italy; ^2^ Metrohm DropSens, Oviedo, Spain

**Keywords:** biosensors, algal toxins, hydrophobin, ScFv, optical detection, electrochemical detection

## Abstract

Although antibody immobilization on solid surfaces is extensively used in several applications, including immunoassays, biosensors, and affinity chromatography, some issues are still challenging. Self-assembling protein layers can be used to coat easily different surfaces by direct deposition. A specific biofunctional layer can be formed using genetic engineering techniques to express fused proteins acting as self-immobilizing antibodies. In this study, fusion proteins combining the self-assembling adhesive properties of a fungal hydrophobin and the functionality of the single chain fragment variables (ScFvs) of two antibodies were produced. The chosen ScFvs are able to recognize marine toxins associated with algal blooms, saxitoxin, and domoic acid, which can bioaccumulate in shellfish and herbivorous fish causing food poisoning. ScFvs fused to hydrophobin Vmh2 from *Pleurotus ostreatus* were produced in *Escherichia coli* and recovered from the inclusion bodies. The two fusion proteins retained the functionality of both moieties, being able to adhere on magnetic beads and to recognize and bind the two neurotoxins, even with different performances. Our immobilization procedure is innovative and very easy to implement because it allows the direct functionalization of magnetic beads with ScFvs, without any surface modification. Two different detection principles, electrochemical and optical, were adopted, thus achieving a versatile platform suitable for different antigen detection methods. The sensitivity of the saxitoxin optical biosensor [limit of detection (LOD) 1.7 pg/ml] is comparable to the most sensitive saxitoxin immunosensors developed until now.

## Introduction

Harmful algal blooms produce a vast quantity of marine toxins, endangering not only the marine world but also human life. These toxins can be accumulated in fish or shellfish (Vilariño et al., 2009), and are often classified according to the human disease for which they are responsible. Saxitoxin (STX) and its derivates, for instance, are classified as paralytic shellfish toxins and can interact with the voltage-gated sodium channels of nerves and muscle cells (Cusick and Sayler, 2013). Moreover, domoic acid (DA) stimulates the glutamate receptors causing amnesic shellfish poisoning (Berman et al., 2002). Therefore, to decrease concerns in terms of human health, environmental preservation, and economic challenges, the detection of algal toxins has drawn attention. To achieve this aim, one of the first methods approved for the study of these toxins was the use of a mouse bioassay. However, due to the unethical nature of animal testing, this technique has been replaced by other analytical techniques, such as HPLC and UPLC-MS/MS (Greer et al., 2016). Nevertheless, these analyses are expensive and sometimes time-consuming, and thus the development of a rapid and specific assay has become necessary. Thanks to antibody/antigen recognition, the use of immunosensors has enabled the design of specific, faster, and cheaper analytical methods for toxin detection. Moreover, the availability of very sensitive biosensors can allow the on-site detection of neurotoxin traces avoiding demanding pretreatments, thus enabling seafood poisoning prevention. Nonetheless, the development of this kind of biosensor is subject to certain critical issues, such as the stability or immobilization of the antibody onto the surface and the right orientation of the antigen-binding site. Considering that the nature and the length of the linker, responsible for the antibody immobilization, affect the antigen recognition, several adhesion methods have been optimized (Jung et al., 2008). Chemical derivatization of the surfaces, followed by covalent attachment, together with the chemical modification of the antibodies by oxidation of the oligosaccharide moiety of the Fc region has been utilized. Likewise, a more biocompatible strategy to obtain a good steric accessibility of the binding site consists of antibody immobilization on a preformed layer of proteins, such as Protein A and Protein G, and able to interact specifically with the Fc region (Pulido-Tofiño et al., 2000). Most of the issues related to the whole antibody can be overcome using only the active moiety, for example, exploiting antigen-binding fragments (Fab) and single chain fragment variable (ScFv), thanks to their easier structure. In particular, ScFvs consist of small sections of heavy chain (VH) and light chain (VL) joined together by a flexible peptide linker. This simple configuration allows an easier recombinant expression than the full-length immunoglobulin, which is limited by the formation of disulfide bonds in the Fc region (Yusakul et al., 2017). This feature often allows their production in fusion with chemical linkers which improve the immobilization process. The chemical linkers could be replaced by self-assembling proteins, thereby obtaining an innovative immobilization method which does not require a surface modification or covalent bond formation.

Hydrophobins are small proteins typical of filamentous fungi and are described as the most powerful surface-active proteins known due to their self-assembling capability at the hydrophobic–hydrophilic interfaces. Their activity is similar to that of the traditional biosurfactants, but the surface activity of hydrophobins depends only on their characteristic amino acid sequence and the 3D structure (Wösten and de Vocht, 2000). The relative positions of the polar and non-polar amino acids seem quite preserved, determining a hydrophobicity pattern typical of these proteins. Furthermore, eight cysteine residues, which form four disulfide bridges, are well conserved. Hydrophobins can be divided into two main classes based on the different lengths of the inter-cysteine spaces and on the clustering of hydrophobic and hydrophilic groups. Class I hydrophobins assemble into polymeric layers composed of fibrillar structures (Kwan et al., 2006) and are only soluble with strong acid treatments. Furthermore, the soluble forms can polymerize back into rodlets under appropriate conditions. In contrast, class II hydrophobins form layers that can be easily solubilized by organic solvents and detergents (Bayry et al., 2012). Their amphipathic nature makes hydrophobins able to change the physicochemical properties of surfaces, for example, rendering hydrophobic surfaces hydrophilic (Wösten et al., 1994). The formed layer can adhere to different surfaces (polystyrene, silicon, glass, and 2D nanomaterials) by direct deposition, allowing the attachment of biomolecules in their active form on these surfaces (Laaksonen et al., 2010; Gravagnuolo et al., 2015; Longobardi et al., 2015; Piscitelli et al., 2017a). A homogeneous biocatalytic layer can also be formed using genetic engineering techniques to produce fused proteins for the development of self-immobilizing chimerae. The class I hydrophobin Vmh2, produced by the fungus *Pleurotus ostreatus*, has been recombinantly expressed fused to a wide range of proteins of biotechnological interest such as the glutathione-S-transferase (Piscitelli et al., 2017c), a multicopper oxidase (Sorrentino et al., 2019), the green fluorescent protein (Piscitelli et al., 2017b), and the human antimicrobial peptide LL-37 (Sorrentino et al., 2020).

Therefore, in this study, fusion proteins have been produced by combining the self-assembling adhesive properties of the Class I hydrophobin Vmh2 and the functionality of the ScFvs of two antibodies against the two marine neurotoxins, STX and DA. Once the refolding procedure had been optimized, the functionality of both fusion partners was verified by immobilizing them on magnetic beads (MBs), without any kind of derivatization, and testing the antigen-binding capability.

## Materials and Methods

All the chemicals used in this study were of analytical grade. All the solutions were prepared using ultrapure water of 18 MΩ cm resistivity (Milli-Q purification system Millipore, Merck, Darmstadt, Germany). Sodium chloride, the NaCl Trizma base, EDTA, urea, Triton X, DTT, isopropyl-β-d-thiogalacto-pyranoside, l-Arginine, and bovine serum albumin (BSA) were purchased from Sigma-Aldrich (Saint Louis, Missouri, United States). Tetramethylbenzidine (TMB), H_2_O_2_, H_2_SO_4_, HRP-DA, and HRP-STX were provided by the ELISA kit Saxitest and Domotest (Zeulab, S.L., Zaragoza, Spain). 2,2′-azino-bis (3-ethylbenzothiazoline-6-sulfonic acid (ABTS) and NaH_2_PO_4_ were purchased from Merck (Darmstadt, Germany).

### Vector Construction

**FIGURE 1:**
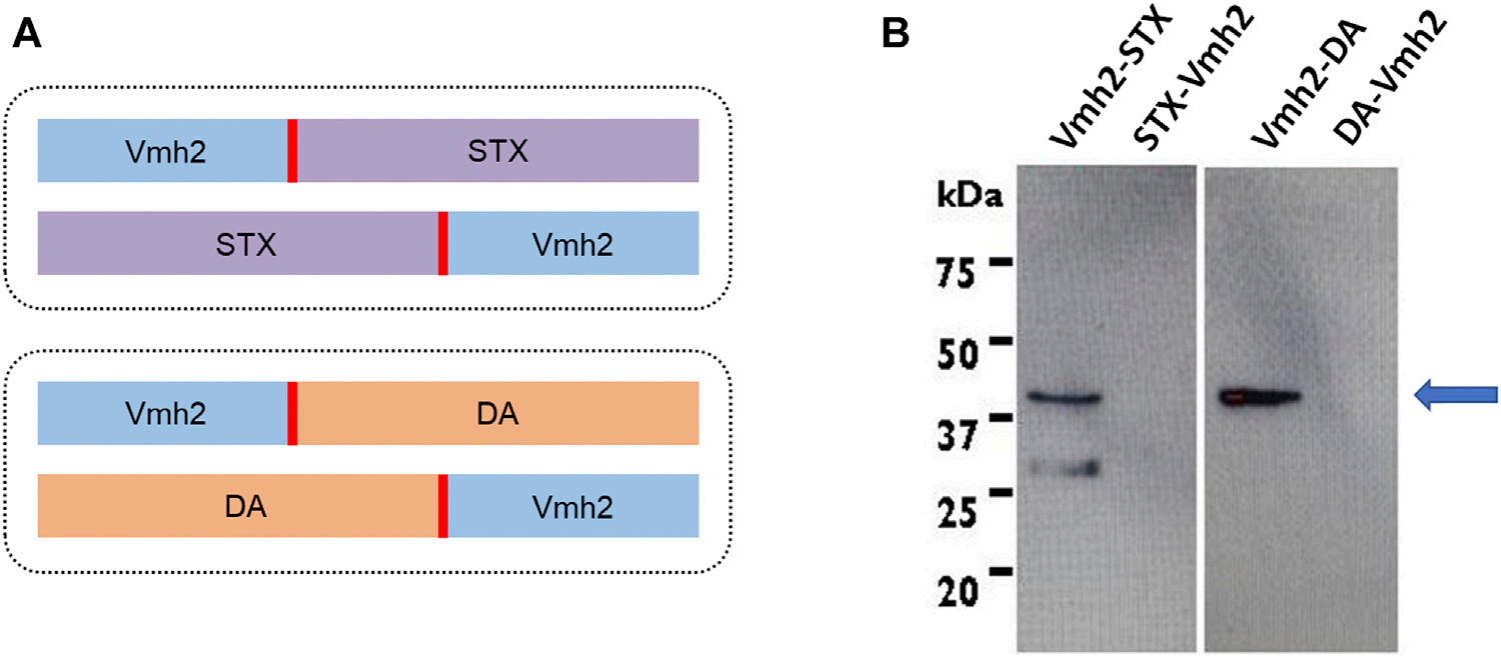
**(A)** Schematic representation of gene constructs [in blue, the hydrophobin; in red, the (Gly_4_Ser)_3_ linker; in violet, the ScFv against saxitoxin; and in orange, that ScFv against domoic acid]; **(B)** Western blotting analysis of the *E. coli* inclusion bodies. The arrow indicates the expected molecular weight of the chimeras produced.

Four synthetic genes encoding for the hydrophobin Vmh2 from *P. ostreatus*, ScFv against STX or DA, a linker (Gly_4_Ser)_3_, and an His-tag were designed, as shown in Figure 1A , and optimized according to the *Escherichia coli* codon usage. The genes obtained were restricted with NcoI and BamHI and ligated into the corresponding sites of the pET22b (+) vector. *E. coli* BL21 (DE3) cells were transformed with the recombinant plasmids pET22b_Vmh2-ScFv_STX_, pET22b_ScFv_STX_-Vmh2, pET22b_Vmh2-ScFv_DA_, and pET22b_ScFv_DA_-Vmh2.

### Expression and Isolation of Chimeric Proteins

For each plasmid transformed, a recombinant colony was transferred from the agar plate to 20 ml of LB medium (1% tryptone, 0.5% yeast extract, 1% NaCl) completed with 100 μg/ml of ampicillin and incubated overnight at 37°C, 180 rpm. The culture was diluted at an OD_600_ value of 0.08 with 200 ml of LB, supplemented with the same antibiotic, and further incubated at 37°C. To induce the recombinant protein expression, 1 mM IPTG (isopropyl-β-d-thiogalacto-pyranoside) was added when the culture reached an OD_600_ value of 0.5. After 3 h of induction, cells were collected by centrifugation at 3,300 × *g* at 4°C for 15 min. The obtained pellet was resuspended in a lysis buffer (100 mM Tris HCl, 10 mM EDTA, 2 M urea, and 2% Triton X-100 pH 8.0), at a final concentration of 20 OD/ml, and subjected to a sonication process (Bandelin, HD3200, MS 72 probe, running at 40% amplitude) for 30 min (30″ ON and 30″ OFF) in an ice bath. The inclusion bodies were collected by a centrifugation step at 3,300 × g for 15 min and then washed three times with the lysis buffer to remove contaminants. Next, the pellets of the inclusion bodies were dissolved in a denaturing buffer (100 mM Tris HCl, 10 mM EDTA, 8 M urea, and 10 mM DTT, pH 8) and incubated for 1 h at 37°C under stirring. The supernatants were collected after centrifugation at 3,300 × *g* at 4°C for 15 min.

### Western blot Analysis

To localize the recombinant proteins, a Western blot analysis was carried out by exploiting the presence of the His-tag at the C-terminus of each chimera. Protein samples were loaded on SDS-PAGE (12.5%) and transferred to a PVDF membrane using an electroblotting transfer apparatus [Trans-Blot Semi-Dry Transfer Cell, Bio-Rad, Segrate (MI), Italy]. The protein detection was carried out by using a monoclonal peroxidase–conjugated anti-polyHistidine antibody at a 1:2,500 ratio (Sigma-Aldrich, Saint Louis, Missouri, United States). The membranes were developed by using a chemiluminescent substrate WESTAR ƞC 2.0 (Cyanagen).

### Refolding of Recombinant Vmh2-ScFv_STX_ and Vmh2-ScFv_DA_

The refolding protocol of the inclusion bodies was divided into two main steps: i) ultrafiltration and dialysis against the refolding buffer (50 mM Tris HCl pH 8, 1 mM EDTA, 150 mM sodium chloride, 500 mM l arginine, and 2 mM of DTT) to dilute the urea up to a concentration of 0.8 M using a Centricon centrifugal filter unit with a polyethersulfone (PES) membrane (cutoff of 10 kDa); and ii) after an overnight storage at 4°C, dilution of l-Arginine up to a concentration of 50 mM through ultrafiltration and dialysis against 50 mM Tris HCl pH8 + 0.8 M urea. The protein concentration was evaluated using the Pierce 660 nm Protein Assay Kit (Thermo Scientific, Waltham, MA) using bovine serum albumin (BSA) as standard protein.

### Chimera Immobilization on Magnetic Beads

10 mg of pristine MBs (Absolute Mag™ Magnetic Particles, 3.0–3.9 μm, Creative Diagnostics, paramagnetic particles prepared by coating a layer of iron oxide and polystyrene over polystyrene core particles) were washed once with 1 ml of water and three times with 1 ml of 50 mM Tris HCl pH8 + 0.8 M urea. Next, the MBs were incubated with 50 µg of Vmh2-ScFv_STX_ or Vmh2-ScFv_DA_ on a rotary tube mixer for three days at 4°C. The functionalized MBs were collected by using a magnet and after three washes with buffer, the immobilization yields were calculated as a difference between the incubated protein amount and that measured in the supernatant and in the washes.

Vmh2-ScFv_STX_ and Vmh2-ScFv_DA_ were also incubated as previously described, but in the absence of the MBs, to rule out protein adhesion on the walls of the reaction vials.

### Antibody/Vmh2-ScFv_STX_ Competition

A competitive assay between the anti-STX antibodies of the STX ELISA kit and the chimera Vmh2-ScFv_STX_ was carried out. To achieve this aim, the ELISA test kit for STX detection (SAXITEST) was used in accordance with the manufacturer’s instructions (Zeulab, S.L., Zaragoza, Spain), adding the Vmh2-ScFv_STX_ instead of the antigen standard solution. Thus, 100 μL of the kit dilution buffer, 25 μL of anti-STX antibody solution, 50 μL of STX antigen labeled with horseradish peroxidase (HRP-STX) solution, and 25 μL of different concentrations (0, 20, 40, 50, 60, 80, and 100 ng/ml) of Vmh2-ScFv_STX_ were incubated for 30 min at room temperature in each well (containing immobilized sheep anti-rabbit IgG antibodies). After incubation, the plate was emptied and washed three times with the washing buffer and then dried. Subsequently, 100 μL of the substrate mixture 3,3′,5,5′-tetramethylbenzidine (TMB) and hydrogen peroxide were integrated in each well. The enzymatic reaction was carried out for 15 min at room temperature in darkness and then was terminated by adding100 μL of a 0.5 M H_2_SO_4_ solution. This solution stopped the reaction by decreasing the pH until the HRP lost its activity. Moreover, at this acidic pH, the charge transfer of the complex generated by the oxidation of the TMB yielded to a stable diamine, changing the color of the solution from blue to yellow. Finally, 50 μL of this solution was dropped on the electrode’s surface and a potential of + 0.10 V was applied on the Ag pseudo reference electrode for 60 s.

### Optical Determination of ScFv Functionality

The capability of the ScFvs to bind the corresponding antigen was tested using some elements of two commercial ELISA kits (Saxitest and Domotest, Zeulab S.L., Zaragoza, Spain) and optimizing different parameters, such as volume, time, and temperature of incubation. In detail, 200 µL of functionalized MBs (10 μg of the Scfv chimera and 2 mg of MBs) were precipitated by using a magnetic field, and the supernatant was removed. Next, 200 µL of a labeled toxin, either HRP-STX or HRP-DA at different dilution rates (1:4, 1:10, 1:20), were added to the MBs and incubated for 3 h at 4°C under stirring. As a control, the non-functionalized MBs were subjected to the same treatment, thereby verifying that no toxin was bound to the surface. After incubation, 50 µL of each supernatant sample was assayed in a multiwell plate with 250 µL of 9.1 mM ABTS (2,2′-azino-bis 3-ethylbenzothiazoline-6-sulfonic acid) dissolved into 100 mM NaH_2_PO_4_, pH 5, and 10 µL of 0.3% hydrogen peroxide at 420 nm. Thus, the ScFv-binding capability was calculated as a difference between the activity (mU) in the supernatant of each sample and in the corresponding control. Moreover, a competitive assay was performed on Vmh2-ScFv_STX_ functionalized MBs using the labeled STX (diluted 1–20) and the standard toxin at different concentrations (from 5 to 100 pg/ml).

### Electrochemical Detection of ScFv Functionality

Electrochemical assay conditions were set up, optimizing different parameters, such as volume, time, and temperature of incubation. Aliquots of 100 μL of functionalized MBs (5 μg of ScFv chimera and 1 mg of MBs) were incubated in the presence of 50 μL of DA-HRP/STX-HRP and 50 μL of DA/STX standard solutions at different concentrations under stirring for 45 min at room temperature. Furthermore, an aliquot of 100 μL of functionalized MBs (5 μg of ScFv chimera and 1 mg of MBs) was incubated in the absence of both a labeled and standard toxin to obtain a blank signal. After the incubation reaction, in order to remove all the unbound antigens, the dispersions were subjected to a magnetic separation and washing steps by using 50 mM Tris-HCl buffer solution pH 8 + 0.8 M urea. A screen-printed carbon electrode (SPCE) (DRP-110, Metrohm DropSens, S.L) was positioned onto the magnetic support, and then an aliquot of 25 μL of MB dispersion was deposited onto the electrode surface. After 2 min, all the MBs had been attracted to the working electrode surface. Next, the drop was carefully removed. Subsequently, 20 μL of TMB/H_2_O_2_ stock solution were added and incubated on the electrode in darkness at room temperature for 15 min. Hence, the enzymatic reaction produced the oxidation of TMB by HRP in the presence of H_2_O_2_ generating a blue-colored complex product. Thereafter, the detection took place by the electrochemical reduction of the oxidized TMB applying a potential of −0.20 V vs. the Ag pseudo-reference electrode for 60 s. The reduction current was related to the labeled antigen concentration and inversely related to that of the standard solutions.

## Results

### Production of Vmh2-ScFv_STX_ and Vmh2-ScFv_DA_

Chimeric proteins were designed with the Vmh2 moiety at the N- or C-terminus and linked to the ScFvs through a sequence of 15 aa (Gly_4_Ser)_3_ (Figure 1A). Their production was induced by adding 1 mM IPTG to the culture during the exponential phase. Next, taking advantage of the His-tag at the C-terminus of each chimera, a Western blot analysis using a monoclonal anti-polyHistidine antibody was performed (Figure 1B), showing that only chimeric proteins carrying the Vmh2 at the N-terminus (Vmh2-ScFv_STX_ and Vmh2-ScFv_DA_) were detectable and localized in the inclusion bodies.

After solubilization of the inclusion bodies in 8 M urea, an *in vitro* refolding procedure was set up. The optimized protocol consisted of two ultrafiltration steps in which the presence of l-Arginine (Lange and Rudolph, 2009) and 0.8 M urea prevented the chimera aggregation. Nonetheless, the l-Arginine was then removed by dialysis to avoid its interference with the following characterizations. The production yield of both the refolded Vmh2-ScFv_STX_ and Vmh2-ScFv_DA_ was about 5 mg per liter of culture broth. However, other strategies could be adopted to improve the amount of the recombinant protein produced, as suggested by Kirkland et al. (Kirkland and Keyhani, 2011). The refolding procedure was tested, verifying both the adhesive capability of the hydrophobin Vmh2 and the antigen-binding ability of both ScFvs, as described below.

### Characterization of Fusion Proteins

#### Vmh2-ScFv_DA_

**FIGURE 2:**
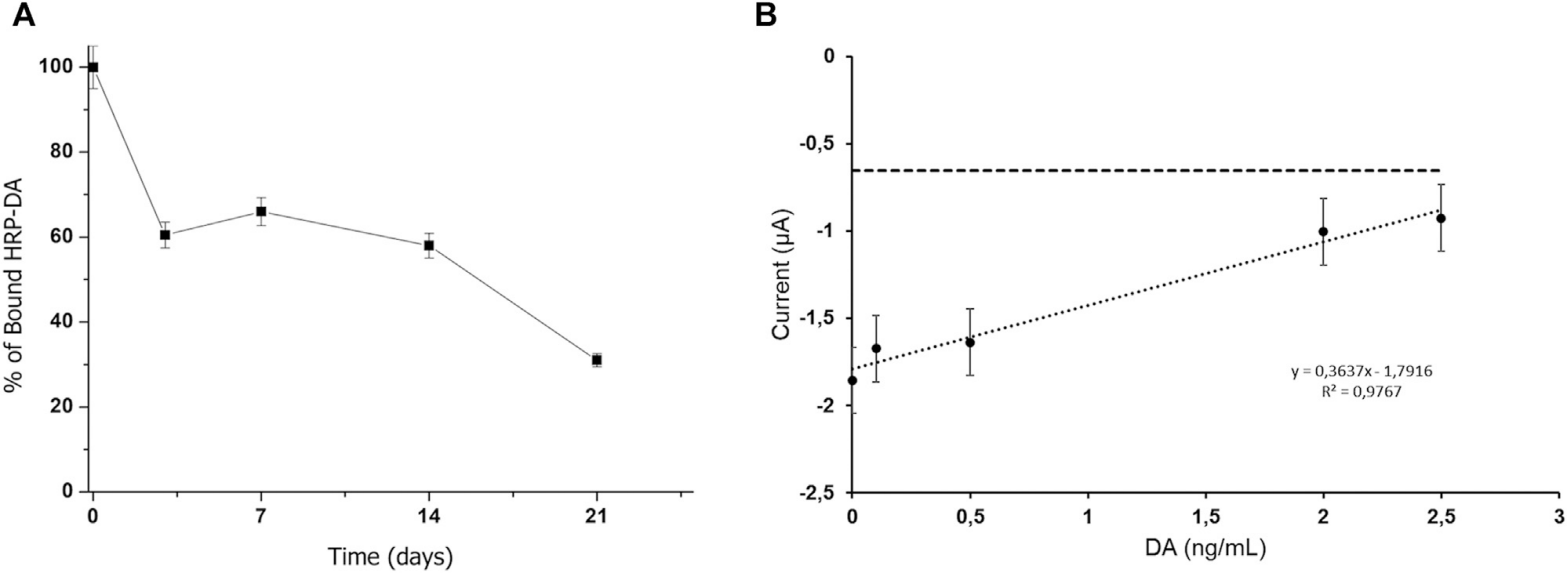
**(A)** Stability of the Vmh2-ScFv_DA_–functionalized MBs, **(B)** electrochemical detection of the competitive assay using Vmh2-ScFv_DA_–functionalized MBs with the DA concentration in the range from 0 to 2.5 ng/ml. The horizontal line indicates the electrochemical background signal.

The functionality of the adhesive moiety of Vmh2-ScFv_DA_ was verified by incubating 50 μg of the chimera with 10 mg of pristine MBs. No protein adhesion on the walls of the reaction vials and no protein in the solution were detected, thus indicating successful protein immobilization on the beads. On the other hand, when BSA was used as the control, 38 μg of the protein were still present in the solution. Furthermore, Vmh2-ScFv_DA_ was tightly bound to the magnetic surface even after at least three washes with the buffer. Additionally, the capability of Vmh2-ScFv_DA_ to bind the toxin was verified using a colorimetric method based on DA labeled with peroxidase. In the optimized conditions (chimera/MB ratio 0.005 w/w and HRP-DA solution diluted 1:20), the amount of captured HRP-DA was 0.20 ± 0.05 mU/mg MBs, as calculated by the difference between the peroxidase activity measured in the sample supernatant before (0.9 mU/mg MBs) and after incubation. This value represents a clear indication of a successful chimera/toxin interaction. Nonetheless, a decrease in this binding capacity was observed over time, reducing to a quarter of the initial-binding capacity after 21 days at 4°C (Figure 2A).

Since the amount of the bound HRP-DA was too low to perform a colorimetric competitive assay with the DA standard solutions, amperometric analyses were carried out tracking the HRP activity electrochemically. Figure 2B shows the current intensity obtained for the calibration curve using DA at concentrations in the range from 0 to 2.5 ng/ml. The electrochemical signal was inversely proportional to the DA concentration, and a linear fitting with a correlation coefficient of 0.9767 was achieved with a calculated limit of detection (LOD) of 0.35 ng/ml, according to 3σ/m (where *σ* is the standard deviation of the estimated intercept and m is the slope of the calibration curves). This trend highlights the competitive reaction between HRP-DA and DA for the binding domain of the ScFv moiety of the chimera and confirms the successful interaction between the Vmh2-ScFv_DA_ fusion protein and the antigen.

#### Vmh2-ScFv_STX_

In the case of the chimera Vmh2-ScFv_STX_, an electrochemical analysis aimed at evaluating the competition between the free Vmh2-ScFv_STX_ chimera and the commercial antibody against STX was successfully performed.

**FIGURE 3:**
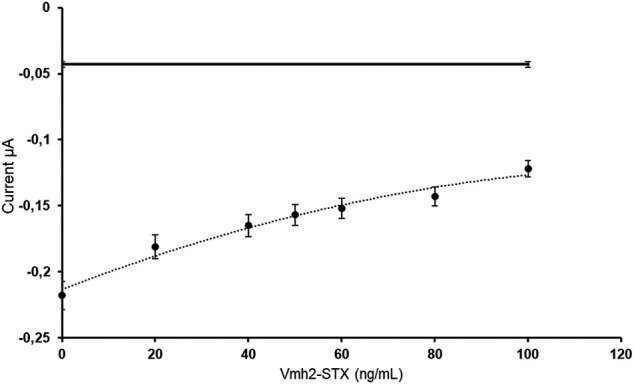
Electrochemical measurements of the free Vmh2-ScFv_STX_/anti-STX competitive assay with the dashed line as an eye-guide. The horizontal line indicates the electrochemical background signal.

The ELISA SAXITEST was performed by adding Vmh2-ScFv_STX_ at different concentrations to a constant amount of the HRP-STX and anti-STX antibodies. In this way, the anti-STX antibodies bound to the wells were modified with the secondary antibodies, while Vmh2-ScFv_STX_ was removed during washing. Therefore, an increasing concentration of the free chimera would produce a decrease in the anti-STX/HRP-STX complex with a consequent decrease of the electrochemical signal. Figure 3 shows the currents obtained for each concentration of free Vmh2-ScFv_STX_, indicating that the chimera really competes with the commercial antibody and a 50% inhibition was achieved at a concentration value of 60 ng/ml.

**FIGURE 4:**
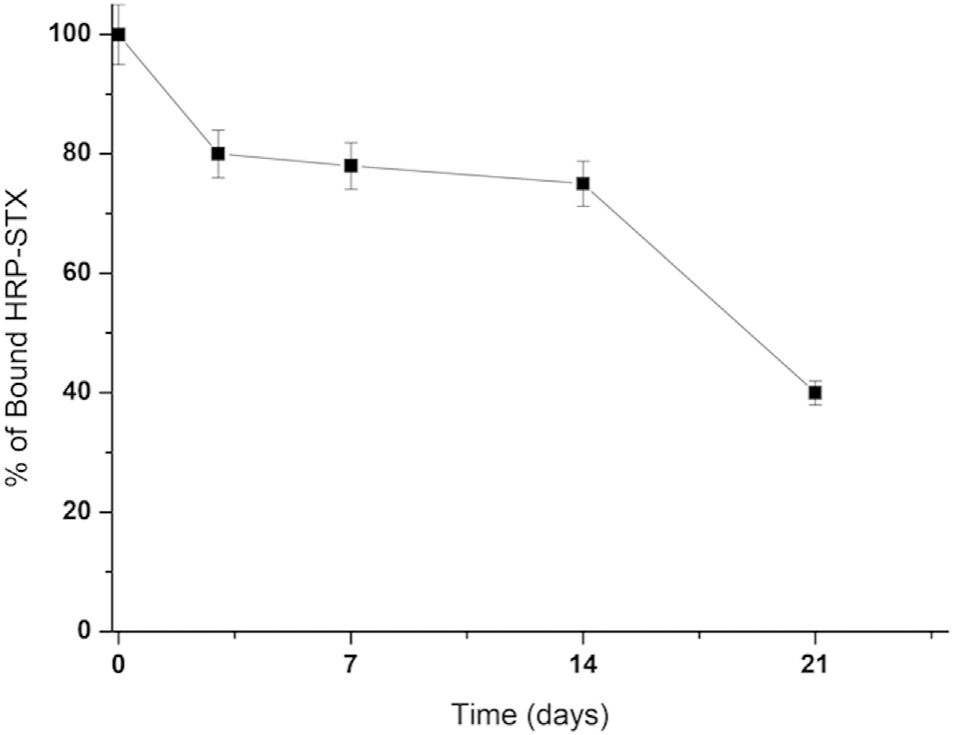
Stability of the Vmh2-ScFv_STX_–functionalized MBs.

As already observed for Vmh2-ScFv_DA_, the immobilization of Vmh2-ScFv_STX_ on pristine MBs was successful, indicating the proper functionality of the hydrophobin moiety. To verify the functionality of the ScFv moiety upon immobilization, the MBs functionalized with the chimera were incubated with labeled STX (HRP-STX, diluted 1:20, 3.6 mU/mg MBs) and analyzed by the HRP colorimetric test. The amount of captured HRP-STX was 0.50 ± 0.05 mU/mg MBs, confirming the efficient antigen/antibody interaction. Moreover, 40% of its functionality was retained after 21 days at 4°C (Figure 4).

The functionalized MBs were exploited to perform competitive assays between STX at different concentrations and HRP-STX, using both optical and electrochemical measurements.

**FIGURE 5:**
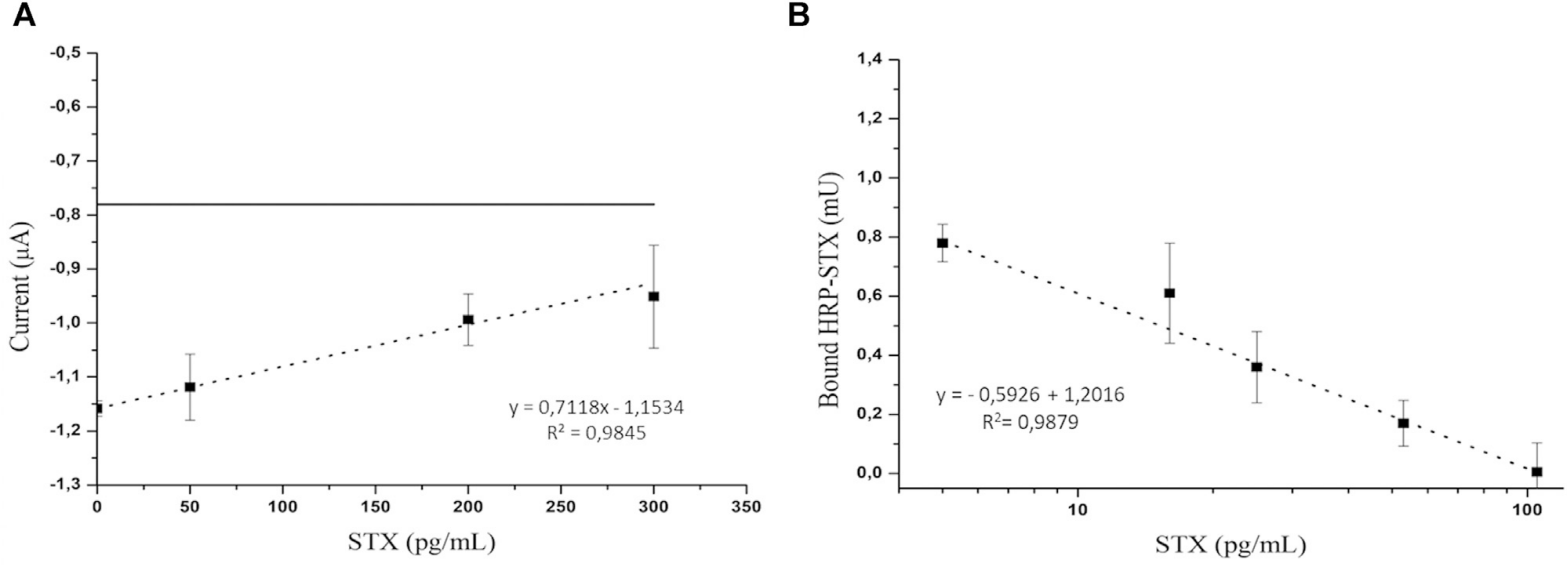
**(A)** Electrochemical detection of the competitive assay using a STX concentration in the range from 0 to 300 pg/ml. The horizontal line indicates the electrochemical background signal; **(B)** optical detection of the competitive assay using an STX concentration in the range from 0 to 100 pg/ml.

As can be seen in Figure 5A, which reports the electrochemical measurements, a linear trend (correlation coefficient of 0.9845) was observed in the concentration range from 0 to 300 pg/ml, obtaining an LOD of 52 pg/ml and RSD (relative standard deviation) (*n* = 3) = 9.57%.

In the optical platform, a linear trend (correlation coefficient of 0.9879) (Figure 5B) of the HRP activity as a function of the logarithm of STX concentration was observed in the concentration range from 0 to 100 pg/ml, obtaining an LOD of 1.7 pg/ml.

## Discussion

Although surface modifications are often the best way to immobilize antibodies, this process can be expensive in terms of both time and reagents. A stable protein coating can be formed using a self-assembling protein layer and/or its engineered variants. Indeed, chimeric proteins formed by adhesive and recognition moieties may represent a breakthrough in the field of surfaces functionalization. Herein, with the aim of enabling antibody immobilization without any chemical derivatization, we have developed for the first time a device based on the fusion of a class I hydrophobin with proteins able to bind antigens, ScFvs. In particular, the adhesive capability of the hydrophobin Vmh2 fused to the two ScFvs against the marine toxins STX and DA was exploited to produce adhesive antibodies. Two different combinations of each chimera were designed, with the hydrophobin at the N- or C-terminus of the constructs (Vmh2-ScFv_STX_, Vmh2-ScFv_DA_, ScFv_STX_-Vmh2, and ScFv_DA_-Vmh2, respectively) (Figure 1A). All the fusion proteins were cloned and produced in *E. coli*, but only the Vmh2-ScFv_STX_ and Vmh2-ScFvDA chimeras were detected. This result could be explained in terms of a different stability at the transcript levels or a different propensity to aggregation of the proteins. Indeed, Vmh2-ScFv_STX_ and Vmh2-ScFv_DA_ were found localized in the inclusion bodies, as that occurred in the case of the other Vmh2 chimeras previously produced in *E. coli* (Piscitelli et al., 2017c; Puopolo et al., 2021), with harsh conditions being necessary to solubilize them. According to the TANGO algorithm (tango.crg.es), ScFv_DA_-Vmh2 and ScFv_STX_-Vmh2 have shown a higher propensity for amyloid aggregation. Therefore, their solubilization could be even more difficult, rendering their detection unachievable. In our experiments, the setting up of a refolding procedure was a crucial step in the whole process and needed to be validated by evaluating the functionality of both moieties. The adhesiveness of the chimeras was easily ascertained although assessing the binding capacity of the ScFvs was trickier.

Vmh2-ScFv_STX_ and Vmh2-ScFv_DA_ were immobilized on pristine MBs since MBs are often used to develop optical or electrochemical immunosensors, thanks to their own characteristics. They show a large surface to the volume ratio, a wide surface area, and, most importantly, they can be recovered using a magnetic field (Bilal et al., 2018), allowing their usage as a carrier to pre-concentrate the analytes and to enhance the sensitivity of the systems (Toldrà et al., 2021). Both chimeras were almost completely immobilized on this surface without the need of any chemical derivatization. Concerning the functionality of the ScFvs moiety, a different behavior between the two fusion proteins was observed. Vmh2-ScFv_DA_ bound a lower amount of the labeled toxin and was less stable, with respect to Vmh2-ScFv_STX_, as tested by the optical assay. This difference could be due to an unsatisfactory refolding process of Vmh2-ScFv_DA_, making the optical competitive assay unachievable. On the other hand, the competitive assay was accomplished using the electrochemical technique. Indeed, the calculated LOD (0.35 ng/ml) was higher than that (0.1 ng/ml) described by Kania et al. (2003), while it was lower than that recently developed by Nelis et al. (2021), whose LOD is 1.7 ng/ml.

A more complete characterization of the antigen-binding capability was achieved in the case of Vmh2-ScFv_STX_. This chimera was able to compete in solution with a commercial antibody against STX, thus confirming its functionality in STX binding. Both optical and electrochemical detection were used to measure STX in a competitive assay obtaining very low LODs, 1.7 pg/ml and 52 pg/ml, respectively. The sensitivity of the optical platform is comparable to that (1.2 pg/ml) recently obtained by Jin et al. (2019), who developed an ultrasensitive immunosensor to detect traces of STX based on a magnetic gold electrode, antibody-functionalized MBs, and palladium-doped graphitic carbon nitride nano-sheets, which possess a peroxidase-like activity. Even the analyte concentration range of our electrochemical detection (0–300 pg/ml) is comparable to that obtained by Jin et al. (2019) (0–400 pg/ml). On the other hand, our immobilization procedure is innovative, very easy to implement, and smart, allowing the direct functionalization of MBs with ScFvs.

## Data Availability

The original contributions presented in the study are included in the article further inquiries can be directed to the corresponding author.
